# Association Between Infant-Feeding Practices and Early Childhood Caries: A Clinical and Epidemiological Study

**DOI:** 10.3390/children12121697

**Published:** 2025-12-16

**Authors:** Krisztina Martha, Orsolya Kovács, Csaba Dudás, Henrietta Dudás, Esztella-Éva Kis

**Affiliations:** Faculty of Dental Medicine, George Emil Palade University of Medicine, Pharmacy, Science and Technology of Târgu Mures, 38 Gh. Marinescu Str., 540139 Târgu Mureș, Romania; krisztina.martha@umfst.ro (K.M.); kovacslia2000@gmail.com (O.K.); maria-henrietta.dudas@umfst.ro (H.D.); esztella.kis@umfst.ro (E.-É.K.)

**Keywords:** early childhood caries (ECC), breastfeeding, dmft index

## Abstract

**Highlights:**

**What are the main findings?**

- This study shows that children who were breastfed had significantly lower dmft scores compared with those who were mixed- or formula-fed, indicating a protective effect of breastfeeding against early childhood caries (ECC).
- A longer duration of breastfeeding and daily use of fluoride toothpaste were identified as independent protective factors against caries in the logistic regression analysis.

**What are the implications of the main findings?**

- Promoting breastfeeding and providing appropriate oral hygiene education during infancy may help reduce the risk of ECC and improve long-term oral health outcomes.
- Incorporating guidance on infant feeding and daily fluoride toothpaste use into pediatric care and community health programs could strengthen early prevention efforts.

**Abstract:**

**Background**: Early childhood caries (ECC) remains one of the most prevalent chronic conditions among preschool children worldwide. Feeding practices during infancy play a significant role in shaping oral microbial colonization and caries risk. This study aimed to evaluate the association between breastfeeding, artificial feeding, and the occurrence of ECC among children aged 3–5 years. **Methods**: A cross-sectional clinical and epidemiological study was conducted involving 103 children aged 3–5 years. Parents completed a structured questionnaire. Participants were divided into three groups based on infant feeding history: exclusively breastfed, exclusively artificially fed, and mixed-fed. Clinical oral examinations assessed dmft indices. Statistical analysis included Kruskal–Wallis and Mann–Whitney U tests for dmft scores, Chi-square tests for categorical variables, Spearman correlations, and binary logistic regression to identify predictors of dental caries. **Results**: Among participants, 43.6% were exclusively breastfed, 41.7% mixed-fed, and 14.5% exclusively artificially fed. The mean dmft index was highest in formula-fed children (4.2 ± 3.78), followed by mixed-fed (2.97 ± 3.19) and breastfed children (1.75 ± 2.99). Kruskal–Wallis analysis showed significant differences in dmft among groups (*p* = 0.005), with breastfed children having lower dmft than both formula-fed (*p* = 0.009) and mixed-fed (*p* = 0.006) children. Caries presence was significantly associated with feeding type (χ^2^ = 14.00, *p* = 0.001) and fluoride toothpaste use (χ^2^ = 7.56, *p* = 0.023). A weak negative correlation was observed between dmft and breastfeeding duration (ρ = −0.266, *p* = 0.007). Logistic regression identified longer breastfeeding duration (OR = 0.889, 95% CI: 0.81–0.97, *p* = 0.010) and use of fluoride toothpaste (OR = 0.323, 95% CI: 0.13–0.81, *p* = 0.012) as protective factors against dental caries. Parental questionnaire responses suggested prolonged bottle feeding and nocturnal feeding habits as contributing factors. **Conclusions**: Longer breastfeeding duration and regular use of fluoride toothpaste were associated with lower risk of dental caries in children. Formula feeding was associated with higher dmft scores. Infant feeding practices significantly influence the risk of early childhood caries. Encouraging breastfeeding and educating parents on appropriate weaning and oral hygiene measures may reduce ECC incidence and support better long-term oral health outcomes.

## 1. Introduction

Early childhood caries (ECC) represents a major global public health problem, particularly in low- and middle-income countries, where its prevalence can exceed 60% among preschool-aged children [1]. We are dealing with a pervasive and persistent global health challenge, affecting millions of preschool-aged children and imposing a substantial burden on families, healthcare systems, and communities. Despite improvements in oral health awareness and preventive strategies, ECC remains one of the most prevalent chronic diseases of childhood, the term of “social disease” can easily be used. The WHO (World Health Organization) and numerous epidemiological studies consistently report high ECC rates. Roughly half of all young children are affected, with prevalence rates often around 48–50% in studies using WHO criteria but varying wildly from 1–12% in some developed nations to over 70% in disadvantaged areas or less developed countries, increasing with age, peaking around 3–5 years old, and heavily influenced by sugary diets and socioeconomic status [2].

ECC is a multifactorial disease in which biological, environmental, dietary, behavioral, and socioeconomic determinants interact to influence susceptibility to demineralization and caries progression. Among these determinants, feeding practices in infancy and early childhood have been recognized as key modulators of oral microbiome, salivary physiology, and exposure to fermentable carbohydrates, thus playing a crucial role in shaping early oral health trajectories [3].

Universally recommended infant feeding mode is breastfeeding, due to its well-established nutritional, immunological, psychological and developmental benefits [4]. Human milk contains lactose as its primary carbohydrate source; although lactose is fermentable, it is inherently less cariogenic than sucrose-rich alternatives commonly found in formula, sweetened beverages, or complementary foods [5]. More importantly, human milk delivers an array of protective components—including secretory immunoglobulin A (IgA), lactoferrin, lysozyme, and various bioactive peptides [6]—that modulate the oral microbiome and inhibit colonization by cariogenic organisms such as *Streptococcus mutans*. These protective factors, combined with the mechanical stimulation of oral tissues, perioral muscular activity and enhancement of salivary flow during suckling, contribute to an environment generally unfavorable for early cariogenic activity [7]. However, evidence also indicates that certain feeding patterns—particularly prolonged or nocturnal breastfeeding after tooth eruption, when salivary flow is physiologically reduced—may increase caries risk under specific conditions, especially when not accompanied by adequate plaque control or fluoride exposure [8].

Formula feeding and mixed feeding practices introduce different patterns of carbohydrate exposure and feeding behaviors. Many commercial formulas contain fermentable sugars with higher cariogenic potential than lactose, and bottle-feeding habits such as prolonged or ad libitum (on-demand) nighttime feeding may prolong oral clearance time, creating an acidic environment conducive to enamel demineralization [9]. Additionally, early introduction of complementary foods, routine consumption of sugar-containing beverages, and suboptimal oral hygiene practices may further amplify caries risk. Recent epidemiological studies have therefore emphasized the importance of evaluating not only the type of feeding (breast feeding, formula feeding, or mixed feeding) but also the duration, frequency, nighttime feeding patterns, and the broader behavioral context in which feeding occurs [10,11].

Understanding the association between infant feeding practices and ECC is crucial for formulating evidence-based preventive strategies within pediatric dentistry and public health [12]. Several investigations have shown that breastfed children tend to present with lower dmft scores compared with bottle-fed or mixed-fed children, suggesting a protective effect of breastfeeding. Conversely, formula feeding and prolonged bottle use—particularly when associated with sweetened liquids—have been linked with earlier colonization by cariogenic bacteria, increased plaque accumulation, and greater ECC prevalence [10]. Yet, ambiguities remain due to variations in study design, population characteristics, and confounding behavioral factors such as oral hygiene habits, fluoride use, and family dietary patterns [13,14].

Given these complexities, ongoing research aims to clarify the independent contribution of infant feeding practices to ECC risk while accounting for behavioral modifiers. The current study builds on this effort by examining feeding patterns and caries outcomes in a cohort of children aged 3–5 years, incorporating clinical findings, detailed parental questionnaires, and relevant sociodemographic factors. As highlighted in the preliminary data, breastfeeding may confer a protective effect, while formula feeding and mixed feeding appear more frequently associated with higher dmft scores.

Investigating these relationships is essential not only for guiding parental counseling but also for informing public health policies that integrate oral health recommendations into early childhood nutrition programs.

To systematically evaluate the relationship between feeding modality and caries experience in the primary dentition, the following hypotheses were formulated: (H_0_) there is no significant difference in caries prevalence between breastfed and formula-fed children; (H_1_) significant differences exist between feeding groups. By testing these hypotheses, the present study aims to clarify the extent to which early feeding behaviors influence the development of ECC and to identify modifiable factors that may enhance preventive strategies within clinical and community-based pediatric care.

## 2. Materials and Methods

The approval of the study by the Ethics Committee of the “George Emil Palade” University of Medicine, Pharmacy, Sciences and Technology in Târgu Mureș was carried out by decision no. 3685 of 17 March 2025. A cross-sectional study was conducted on a sample of 103 children aged 3–5 years, participants in preschool education in a medium urban area from the central part of Romania. Parents provided written informed consent and completed a structured questionnaire regarding infant feeding habits, duration of breastfeeding or artificial feeding, oral hygiene routines, and dietary behaviors. Subjects were included only after written consent was obtained and the questionnaire was filled totally.

The questionnaire consisted of 29 questions, grouped into six sections. The first four questions revealed demographic data about the examined child, name, sex, date of birth, birth weight and gestational age (born at term or premature). Questions 5–11 were related to breastfeeding, artificial or mixed feeding and duration of their use (in months). Questions 12–15 were those regarding dietary diversification and consumption of certain foods (sweetened drinks, sweets, fruits, dairy products). Questions 16–21 were about the beginning and frequency of oral hygiene, materials used and parents’ opinion on the importance of oral health. Chapter 5 consisted of questions 22–25 related to family history, frequency of caries in the family and parents’ habits regarding oral hygiene. Questions 26–29 related to the parent’s awareness of the child’s oral health, the number of teeth affected by dental caries, periodic check-ups and fluoride treatments. The last chapter was dedicated to additional observations related to the topic and it was in fact an open-ended question for any observations from the parents for the examiner.

Clinical dental examinations were performed by calibrated examiners under standardized lighting, following World Health Organization (WHO) criteria for caries diagnosis. The decayed, missing and filled teeth (dmft) index was recorded according to WHO conventions: the “m” (missing) component was recorded only when there was clear clinical or historical evidence that the primary tooth was lost due to caries. Teeth not yet erupted were not counted as missing. Participants were categorized into three feeding groups based on their primary feeding type during infancy: Breastfed (*n* = 45), Artificially fed (formula-fed) (*n* = 15), and Mixed feeding (*n* = 43).

Data was analyzed using IBM SPSS Statistics version 25 (IBM Corp., Armonk, NY, USA). Normality of the dmft variable was tested using the Shapiro–Wilk test, which indicated that the dmft scores in the breastfed and mixed feeding groups were not normally distributed, whereas the artificially fed group showed a normal distribution. Because the assumption of normality was violated, a non-parametric Kruskal–Wallis test was used to compare dmft scores among the three feeding groups. When the Kruskal–Wallis test indicated a statistically significant difference, pairwise post hoc comparisons were conducted using the Mann–Whitney U test. To control for multiple testing, a Bonferroni correction was applied.

Associations between feeding type and categorical variables were evaluated using the Chi-square (χ^2^) test of independence. The strength of significant associations was estimated using Cramer’s V. The significance level was set at *p* < 0.05.

The relationship between continuous variables was evaluated using the Spearman rank-order correlation test, as the dmft index was not normally distributed according to the Shapiro–Wilk test. Correlations were computed between dmft and age, breastfeeding duration (month), and mean birth weight. Correlation coefficients (ρ) were interpreted as follows: values of 0.00–0.19 = very weak, 0.20–0.39 = weak, 0.40–0.59 = moderate, 0.60–0.79 = strong, and 0.80–1.00 = very strong correlation. Statistical significance was established at *p* < 0.05.

Multivariable analysis was performed to identify independent predictors of dental caries. The dependent variable was caries presence, dichotomized as 0 = caries-free (dmft = 0) and 1 = caries-present (dmft > 0). Independent variables included age, breastfeeding duration (months), mean birth weight, prematurity (<37 weeks), daily sweets consumption, daily sweetened drink consumption, fluoride toothpaste use, and brushing frequency (≥2×/day). A binary logistic regression analysis was performed, and results were expressed as odds ratios (ORs) with 95% confidence intervals (CIs). Model adequacy was assessed using the Omnibus test, Hosmer–Lemeshow goodness-of-fit test, and Nagelkerke R^2^ statistic. A *p*-value < 0.05 was considered statistically significant.

## 3. Results

The interpretation of the questionnaire revealed the following results: Based on the inclusion criteria of the study, the patients studied were divided into three age groups. The results show that 66% of the patients were 5 years old, 21% were 4 years old and 13% were 3 years old. Depending on birth weight, patients were divided into three categories: Under 2500 g at birth (underweight) were 7% of the patients studied. Between 2500 and 4000 g (normal weight) 84% of the patients, and over 4000 g (overweight) were 9% of all studied patients.

Based on gestational age, there were two categories of patients. The first category represents premature patients (<37 weeks) with 7% of the sample. The second category includes those born at term (37–42 weeks), representing 93%.

Regarding the nutrition of patients in the first year of life, three categories were identified: exclusively breastfed (43.6%), mixed fed (41.7%), and exclusively artificially fed (14.5%). Patients exclusively breastfed (45 patients) were divided into two categories: The first category of patients received breast milk until the age of 12 months, 18% of the total. The second category consists of patients breastfed after 12 months, which represents 82% of the total.

According to the duration of exclusive artificial feeding, there were two categories: 27% of patients were artificially fed until the age of 12 months and 73% of patients studied after the age of 12 months. 37% of subjects were breastfed up to 3 months and received formula after the age of one year, 21% of the subjects were breastfed in the first three months and received formula in the first 12 months. 16% of the subjects were breastfed for 6 months and they received formulas starting at the age of one year. Breastfeeding during the first six months followed by formula after that was represented by 7% of the cases. All the other associations between breastfeeding and artificial milk consumption were found in 19% of the cases.

Regarding the introduction of complementary feeding, diversification was introduced in the first 6 months in 28% of cases, between 6–12 months in 71% of cases and in 1% after 1 year of life.

Breastfeeding was considered important by 81% of legal representatives, meanwhile in 32% of the cases artificial feeding is considered important and in the same percentage is less important and is not important for 28% of patients.

Of all participants, 52% did not consume sweetened beverages, and 50% consumed sweets daily, 75% consumed fruit daily, and 68% consumed dairy products daily.

Regarding the distribution of patients based on the first brushing, 41% began after the eruption of the first tooth, 37% after 1 year of age, 22% after 2 years of age. Toothbrushing was performed by parents in 16% of cases, by the child alone in 25%, and by both parent and child in 59%. Brushing frequency was once daily in 41%, twice daily in 56%, and less frequently in 3%.

Of all cases studied, 54% did not use fluoride toothpaste, while 49% used it daily. Parents’ opinions on oral hygiene at home and periodic check-ups showed that 84% of cases consider oral hygiene at home to be very important. Regarding periodic check-ups, the results show that in 53% it is very important and in 50% of cases it is important for parents.

The results show that 61% of families had a high frequency of dental caries, while 39% had few or no caries. Among parents, 79% brushed their teeth twice daily or more, and 21% brushed only once daily. Most children (66%) visit the dentist once a year for a regular check-up, while 33% of patients go only when dental problems occur.

The results show that in 33 cases (32%), parents were not aware of their children’s dental caries. 13% of patients had 1–2 carious teeth, 9% had 3–4, and 3% had 5–6 teeth with carious lesions, regarding fluoride treatment in the dental office, 96% of patients had never received such treatment.

After questionnaire interpretation clinical examination was performed. In 23% of cases the value of the dmft index was between 1–3, in 20% of cases between 4–6 and in 13% the value was over 6. In the remaining cases the value was 0. The average value of the index was 3. In most cases, dental caries affects both the lower and upper arches at the same time (62%) and the most susceptible teeth are the deciduous molars.

A Kruskal–Wallis test showed a statistically significant difference in dmft scores among the three feeding groups (*p* = 0.005). Pairwise comparisons using the Mann–Whitney U test with Bonferroni correction (adjusted α = 0.0167) revealed that Breastfed children had significantly lower dmft scores (Table 1) compared with both artificially fed (*p* = 0.009) and mixed-fed (*p* = 0.006) children. No significant difference was found between artificially fed and mixed-fed groups (*p* = 0279). The mean dmft index was higher among artificially fed children (4.2 ± 3.78) than among breastfed (1.75 ± 2.99) or mixed-fed children (2.97 ± 3.19).

Table 2 summarizes the associations between feeding type and categorical variables. The Chi-square analysis revealed a statistically significant association between feeding type and caries presence (χ^2^(2) = 14.00, *p* = 0.001, Cramer’s V = 0.369), indicating that caries was significantly more prevalent among artificially fed and mixed-fed children compared to breastfed children. A significant relationship was also found between feeding type and fluoride toothpaste use (χ^2^(2) = 7.56, *p* = 0.023, Cramer’s V = 0.271), with a higher proportion of breastfed children reported to use fluoride toothpaste compared to the other groups.

No statistically significant associations were observed between feeding type and low birth weight (χ^2^(2) = 2.93, *p* = 0.232), prematurity (χ^2^(2) = 2.93, *p* = 0.232), daily sweets consumption (χ^2^(2) = 1.82, *p* = 0.403), sweetened drink consumption (χ^2^(4) = 6.46, *p* = 0.168), or brushing frequency (χ^2^(2) = 0.21, *p* = 0.901). Spearman’s correlation coefficients were calculated (Table 3) to examine the relationship between dmft and selected continuous variables. A significant weak negative correlation was found between dmft and breastfeeding duration (ρ = −0.266, *p* = 0.007), indicating that children who were breastfed for longer periods tended to have lower dmft values. No significant correlations were observed between dmft and age (ρ = 0.094, *p* = 0.343) or birth weight (ρ = 0.045, *p* = 0.653). These findings suggest that longer breastfeeding duration may be associated with a reduced risk of dental caries, while age and birth weight appear unrelated to caries experience in this cohort.

The logistic regression model was statistically significant, χ^2^(8) = 18.09, *p* = 0.021, indicating that the included predictors jointly contributed to the likelihood of caries presence. The model demonstrated good fit (Hosmer–Lemeshow χ^2^(8) = 6.99, *p* = 0.538) and explained approximately 21.6% of the variance in caries occurrence (Nagelkerke R^2^ = 0.216).

Two variables were found to be significant independent predictors of caries (Table 4). Longer breastfeeding duration was associated with lower odds of developing caries (OR = 0.889, 95% CI: 0.81–0.97, *p* = 0.010), and use of fluoride toothpaste was also protective (OR = 0.323, 95% CI: 0.13–0.81, *p* = 0.012). Age, mean birth weight, prematurity, dietary factors, and brushing frequency were not significant predictors (*p* > 0.05). The model correctly classified 67% of children as caries-free or caries-affected.

## 4. Discussion

The analysis of the questionnaire and clinical data provides valuable insights into the feeding practices, oral hygiene habits, and dental health status of the pediatric population included in this study. Subjects were selected from preschool education system in a medium urban area from the central part of Romania. Country-wide dental procedures are covered by the National Health Insurance until the age of 18 and there is no need for periodical dental visits for it. The demographic distribution indicates that most children (66%) were aged 5 years, which corresponds to a developmental stage when the prevalence of early childhood caries (ECC) and mixed dentition-related issues become more evident. Most of the children had normal birth weight (84%) and were born at term (93%), suggesting that perinatal factors such as prematurity or low birth weight did not significantly influence the oral health outcomes in this sample. The same results were emphasized by the meta-analysis conducted by Shi et al., which indicated that preterm increased the risk of ECC significantly; however, low birth weight was not a risk factor for ECC [10]. Some studies revealed a connection between high birth weight and dental caries among preschool children [11].

Nutritional patterns in early childhood play a pivotal role in the development of both general and oral health. In this study, nearly half of the patients (44%) were exclusively breastfed, while 42% received mixed feeding, and 14% were exclusively formula-fed. These proportions indicate that breastfeeding remains the predominant practice, consistent with WHO recommendations advocating exclusive breastfeeding for the first six months of life. However, only 18% of children were breastfed up to 12 months, while 82% continued less than a year. Prolonged breastfeeding beyond one year, especially when associated with nocturnal feeding and inadequate oral hygiene, may increase caries risk, as reported in several studies [12].

The timing of complementary feeding (diversification) was generally appropriate, with 71% of parents introducing solid foods between 6 and 12 months. However, 28% of children were diversified before the age of 6 months, which could reflect a lack of adherence to international guidelines. Early diversification, combined with inappropriate feeding habits such as the introduction of sweetened beverages and frequent consumption of sweets (50% daily), may contribute to increased caries prevalence observed clinically. Scientific early feeding strategies, including limiting sugar intake, ensuring an appropriate timing for complementary feeding, maintaining consistent vitamin D supplementation, and promoting dietary diversity, play a key role in preventing ECC [13].

Parental awareness and attitudes play a crucial role in establishing preventive oral health behaviors. Most parents (81%) recognized the importance of breastfeeding, while opinions about artificial feeding were divided. Encouragingly, 84% considered home oral hygiene very important, and 53% rated regular dental check-ups as very important, though 33% of children still visited the dentist only when problems occurred.

Tooth-brushing habits showed generally positive trends: 56% of children brushed twice daily, and in most cases (59%), brushing was a shared responsibility between parents and children, suggesting good parental involvement. However, the late initiation of toothbrushing—after one year of age in 37% and after two years in 22%—may contribute to early plaque accumulation and subsequent caries formation. Additionally, only 49% used fluoride toothpaste, and 96% of children had never received professional fluoride application, highlighting a significant gap in preventive care practices. Several studies revealed that less than twice daily tooth-brushing and difficulties performing the procedure during the first preschool years were significant determinants of caries prevalence at the age of 5 years. Health professionals should, therefore, give special attention and assist parents to improve and optimize their toothbrushing behavior during preschool years [14].

The clinical findings revealed that the mean dmft index was 3, indicating a moderate level of caries experience in this population. Approximately 23% of children had a dmf-t between 1–3, 20% between 4–6, and 13% greater than 6, while 44% were caries-free. The distribution pattern—where 62% of lesions affected both arches, predominantly on deciduous molars—corresponds to the typical localization of early childhood caries, reflecting areas of food stagnation and difficult cleaning access. One of the most recent prevalence studies is the one conducted by Peric, which revealed that there is a noticeable trend of increasing numbers of decayed teeth as children progress through their preschool years. It is imperative to take corrective action enhance the existing oral health prevention program in Serbia with the aim of achieving better dental health among preschool children [15].

Notably, 32% of parents were unaware of their child’s carious lesions, emphasizing the need for better parental education and routine dental assessments. The high frequency of caries within families (61%) also suggests possible behavioral or dietary patterns shared among family members, underlining the importance of family-based preventive strategies.

Overall, the results demonstrate that while awareness of oral hygiene importance is relatively high, preventive practices are inconsistently implemented. The combination of frequent sugar consumption, delayed brushing initiation, limited fluoride exposure, and irregular dental visits contributes to the observed caries prevalence. Strengthening parental education regarding the timing of brushing initiation, fluoride use, and the importance of early dental visits (ideally by the eruption of the first tooth) could substantially reduce caries incidence.

Public health initiatives should focus on reinforcing early preventive care, integrating oral health counseling into pediatric visits, and promoting community-based fluoride programs. Further research should explore correlations between feeding duration, sugar intake frequency, and specific caries patterns to design more targeted interventions.

This study reinforces the evidence that infant feeding practices influence the development of early childhood caries. Children who were predominantly bottle-fed presented with a significantly higher caries experience than those who were breastfed. These findings align with previous research suggesting that prolonged bottle feeding, especially with sweetened liquids, contributes to sustained exposure of the teeth to fermentable carbohydrates and prolonged acid challenge [15,16]. In contrast, breastfeeding has been associated with the promotion of beneficial oral microflora and enhanced salivary flow, both of which contribute to oral health [17,18].

The results of this study should be interpreted considering its cross-sectional design, which limits causal inference. Nevertheless, the observed statistical association between artificial feeding and ECC prevalence underscores the need for early preventive interventions and parental education. Public health initiatives focusing on infant feeding counseling and early dental visits could substantially reduce ECC burden. Future research integrating microbiome analysis and longitudinal follow-up is warranted to elucidate the biological pathways linking feeding practices to oral health outcomes.

Most children in the study were breastfed or received mixed feeding during the first year of life, indicating good adherence to recommended feeding practices. However, prolonged breastfeeding beyond 12 months and early diversification (before 6 months) were relatively common and may contribute to increased risk of dental caries if not accompanied by proper oral hygiene [10].

Most parents were aware of the importance of oral hygiene and engaged actively in their children’s toothbrushing routines. Despite this, toothbrushing was often initiated late—after one year of age, in many cases—and less than half of the children used fluoride toothpaste. Furthermore, professional fluoride application was almost nonexistent, highlighting a gap in preventive dental care [19].

The mean dmft index value of 3 indicates a moderate level of caries experience among the studied children. The most affected teeth were deciduous molars, and carious lesions frequently involved both arches. The fact that one-third of parents were unaware of their child’s dental caries underlines the importance of regular dental examinations.

Although most parents considered oral hygiene and dental check-ups important, only two-thirds of children attended preventive dental visits. Parental attitudes were positive but not always reflected in consistent preventive behavior, particularly regarding diet and fluoride use [20].

There is a clear need for educational programs aimed at improving parents’ knowledge and practices regarding children’s oral health. Preventive strategies should emphasize early initiation of brushing, proper fluoride use, healthy dietary habits, and routine dental visits starting with the eruption of the first tooth.

Like any scientific work, our study has its limitations. Although clinical examinations followed WHO diagnostic criteria and examiners were calibrated, some residual misclassification in the dmft components cannot be completely ruled out (for example, occasional uncertainty regarding whether a missing primary tooth was lost due to caries). Our sample included children aged 3–5 years (mean 4.13 ± 0.82 years), an age range in which primary dentition largely erupted. Age showed no significant association with dmft and was included in multivariable models. Any differential effect of tooth eruption on dmft across feeding groups appears unlikely to have meaningfully influenced the results. Future longitudinal studies that record erupted tooth counts and prospectively document reasons for tooth loss would help to reduce this potential source of bias further.

## 5. Conclusions

The study found that breastfeeding was associated with significantly lower prevalence of dental caries in primary dentition, leading to rejection of the null hypothesis and acceptance of the alternative hypothesis. Formula feeding was associated with higher dmft scores. Feeding practices during infancy play a crucial role in determining early oral health outcomes. Strengthening family-based preventive education and increasing access to preventive dental services are essential steps to reduce the burden of dental caries in early childhood.

The study highlights that while awareness of oral health importance is generally high, practical implementation remains insufficient. Public health programs should emphasize breastfeeding promotion and parental awareness of proper feeding and oral hygiene practices as part of comprehensive strategies to prevent early childhood caries.

## Figures and Tables

**Table 1:** Distribution of continuous variables according to feeding type.

6	Breastfed (n = 45) (±SD)	Artificially Fed (n = 15) (±SD)	Mixed (n = 43) (±SD)	Total (n = 103) (±SD)
**Mean age** (years)	3.99 (±0.76)	3.86 (±0.92)	4.36 (±0.78)	4.13 (±0.82)
**Sex** (M/F)	24/21	8/7	22/21	54/49
**Mean dmft**	1.75 (±2.99)	4.2 (±3.78)	2.97 (±3.19)	2.62 (±3.28)
**Median dmft (IQR)**	0 (3)	3 (6)	2 (4.5)	2 (4)
**Breastfeeding duration interval** (months)	6–≥12	0 (no breastfeeding)	0–≥12	0–≥12

**Table 2:** Comparison of categorical variables according to feeding type.

	Breastfed (n = 45)	Artificially Fed (n = 15)	Mixed (n = 43)	Total (n = 103)
Low birth weight (LBW) (<2500 g)	1 (2.2%)	2 (13.3%)	4 (9.3%)	7 (6.8%)
Mean birth weight (±SD)	3431 (±393)	3124 (±681)	3318 (±627)	3339 (±550)
Prematurity (<37 weeks)	1 (2.2%)	2 (13.3%)	4 (9.3%)	7 (6.8%)
Presence of caries (DMFT > 0)	16 (35.6%)	11 (73.3%)	31 (72.1%)	58 (56.3%)
Daily sweets consumption	24 (53.3%)	4 (26.7%)	19 (44.2%)	47 (45.6%)
Daily sweetened-drinks consumption	2 (4.4%)	0	1 (2.3%)	3 (2.9%)
Fluoride toothpaste use	27 (60%)	3 (20%)	19 (44.2%)	49 (47.6%)
Brushing frequency (≥2×/day)	27 (60%)	9 (60%)	25 (58.1%)	61 (59.22%)

**Table 3:** Spearman’s rank-order correlation between dmft and selected continuous variables.

Pair of Variables	Spearman’s Rho (ρ)	*p*-Value
dmft—Age	0.094	0.343
dmft—Breastfeeding duration (months)	−0.266	0.007
dmft—Mean birth weight	0.045	0.653

**Table 4:** Binary logistic regression analysis for predictors of caries presence.

Variable	B	SE	Wald	*p*	OR (Exp B)	95% CI for OR
Age (years)	−0.131	0.289	0.206	0.650	0.88	0.50–1.55
Breastfeeding duration (months)	−0.118	0.046	6.679	0.010	0.889	0.81–0.97
Mean birth weight	0.255	1.214	0.044	0.833	1.29	0.11–15.0
Prematurity (<37 weeks)	0.353	1.188	0.088	0.766	1.42	0.13–15.3
Daily sweets	0.188	1.121	0.028	0.867	1.21	0.13–11.5
Daily sweetened drinks	−0.077	0.343	0.051	0.822	0.93	0.47–1.84
Fluoride toothpaste use	−1.131	0.452	6.275	0.012	0.323	0.13–0.81
Brushing ≥ 2×/day	−0.522	0.460	1.2911	0.256	0.59	0.24–1.47
Constant	2.441	1.316	3.439	0.064	11.48	-

## Data Availability

Data supporting reported results can be found in Supplementary Materials.

## Supplementary Materials

The following supporting information can be downloaded at: https://www.mdpi.com/article/10.3390/children12121697/s1.

## References

[1] Peric T., Markovic E., Markovic J., Petrovic B., Kilibarda B., Vukovic A., Markovic D. (2025). Prevalence of Early Childhood Caries in 3- to 6-Year-Old Children in Serbia: A National Pathfinder Study. Children.

[2] Uribe S., Maldupa I., Innes N. (2021). The global prevalence of early childhood caries: A systematic review with meta-analysis using the WHO diagnostic criteria. Int. J. Paediatr. Dent..

[3] Meyer F., Enax J. (2018). Early Childhood Caries: Epidemiology, Aetiology, and Prevention. Int. J. Dent..

[4] Feldens C.A., Rodrigues P.H., de Anastacio G., Vitolo M.R., Chaffee B.W. (2017). Feeding frequency in infancy and dental caries in childhood: A prospective cohort study. Int. Dent. J..

[5] William R.A. (2012). Lactose cariogenicity with an emphasis on childhood dental caries. Int. Dairy J..

[6] Kim S.Y., Yi D.Y. (2020). Components of human breast milk: From macronutrient to microbiome and microRNA. Clin. Exp. Pediatr..

[7] Duangthip D., Chen K.J., Gao S.S., Lo E.C., Chu C.H. (2020). Early childhood caries: Current evidence, challenges, and future directions. Community Dent. Health.

[8] Suparattanapong P., Chankanka O., Matangkasombut O., Govitvattana N. (2022). Dental caries and associated risk factors in 13-to 18-month-old infants receiving breast or formula milk feeding: A cross-sectional study. Int. J. Paediatr. Dent..

[9] Alexaki F., Kostopoulou M., Koleventi K., Lygidakis N.N. (2025). Does breastfeeding increase the risk of early childhood caries (ECC)? A systematic review. Eur. Arch. Paediatr. Dent..

[10] Shrestha S.K., Arora A., Manohar N., Ekanayake K., Foster J. (2024). Association of breastfeeding and early childhood caries: A systematic review and meta-analysis. Nutrients.

[11] Wang H., Zhang H., Zeng X., Yu J., Jiang Y., Huang L., Zeng X., Chen Q., Da D., Zhang Y. (2024). Association between high birth weight and dental caries at 4–5 years of age: A birth-cohort study. BMC Oral Health.

[12] Cenzato N., Berti C., Cazzaniga F., Di Iasio G., Scolaro A., Maspero C. (2023). Influence of the type of breastfeeding as a risk or protective factor for the onset of malocclusions: A systematic review. Eur. J. Paediatr. Dent..

[13] Behzad F., Nourzadeh M., Rasouli S. (2024). The Prevalence of Early Childhood Caries (ECC) in Iran: An Overview of Recent Findings, Systematic Reviews, and Meta-Analyses. Health Provid..

[14] Maklennan A., Borg-Bartolo R., Wierichs R.J., Esteves-Oliveira M., Campus G. (2024). A systematic review and meta-analysis on early-childhood-caries global data. BMC Oral Health.

[15] Kirthiga M., Murugan M., Saikia A., Kirubakaran R. (2019). Risk factors for early childhood caries: A systematic review and meta-analysis. J. Glob. Health.

[16] Tinanoff N., Baez R.J., Diaz Guillory C., Donly K.J., Feldens C.A., McGrath C., Phantumvanit P., Pitts N.B., Seow W.K., Sharkov N. (2019). Early childhood caries epidemiology, aetiology, risk assessment, societal burden, management, education, and policy: Global perspective. Int. J. Paediatr. Dent..

[17] Dhull K.S., Dutta B., Pattanaik S., Gupta A., Md I., Wandile B., Pattnaik S. (2024). Decoding early childhood caries: A comprehensive review navigating the impact of evolving dietary trends in preschoolers. Cureus.

[18] Shi L., Jia J., Li C., Zhao C., Li T., Shi H., Zhang X. (2020). Relationship between preterm, low birth weight and early childhood caries: A meta-analysis of the case-control and cross-sectional study. Biosci. Rep..

[19] Duan Z., Du C., Tong M., Zeng X., Zhang Y., Shi H. (2025). Association between first 2 years’ feeding practices and early childhood caries: A birth cohort study in Shanghai. BMC Oral Health.

[20] Boustedt K., Dahlgren J., Twetman S., Roswall J. (2020). Tooth brushing habits and prevalence of early childhood caries: A prospective cohort study. Eur. Arch. Paediatr. Dent..

